# Non-Monotonic Complexity of Stochastic Model of the Channel Gating Dynamics

**DOI:** 10.3390/e25030479

**Published:** 2023-03-09

**Authors:** Lukasz Machura, Agata Wawrzkiewicz-Jałowiecka, Monika Richter-Laskowska, Paulina Trybek

**Affiliations:** 1 Institute of Physics, Faculty of Science and Technology, University of Silesia in Katowice, 41-500 Chorzow, Poland; 2 Department of Physical Chemistry and Technology of Polymers, Silesian University of Technology, 44-100 Gliwice, Poland; 3 Łukasiewicz Research Network–Krakow Institute of Technology, The Centre for Biomedical Engineering, Zakopianska Str. 73, 30-418 Krakow, Poland

**Keywords:** ion channels, stochastic dynamics, information entropy

## Abstract

The simple model of an ionic current flowing through a single channel in a biological membrane is used to depict the complexity of the corresponding empirical data underlying different internal constraints and thermal fluctuations. The residence times of the channel in the open and closed states are drawn from the exponential distributions to mimic the characteristics of the real channel system. In the selected state, the dynamics are modeled by the overdamped Brownian particle moving in the quadratic potential. The simulated data allow us to directly track the effects of temperature (signal-to-noise ratio) and the channel’s energetic landscape for conformational changes on the ionic currents’ complexity, which are hardly controllable in the experimental case. To accurately describe the randomness, we employed four quantifiers, i.e., Shannon, spectral, sample, and slope entropies. We have found that the Shannon entropy predicts the anticipated reaction to the imposed modification of randomness by raising the temperature (an increase of entropy) or strengthening the localization (reduction of entropy). Other complexity quantifiers behave unpredictably, sometimes resulting in non-monotonic behaviour. Thus, their applicability in the analysis of the experimental time series of single-channel currents can be limited.

## 1. Introduction

**Figure 1:**
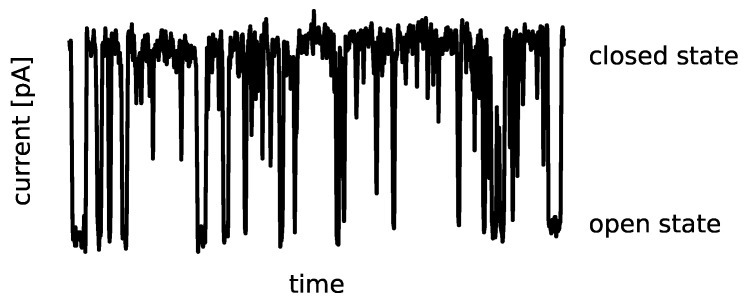
A sample representative of the short excerpt of the experimental signal registered with the patch clamp technique (see [2] for details). The recording has the form of the time series of the single-channel current at fixed external conditions (temperature, membrane potential, solutions, pressure, etc.). Based on the ionic current amplitude, the functional states of the channel can be recognized as open (conducting) and closed (non-conducting). The typical ionic current amplitudes are several to tens of picoamperes. The maximal residence times in open and closed times most frequently do not exceed tens of milliseconds.

Ion channels are transmembrane proteins that fluctuate between variant open (ion-conducting) and closed (non-conducting) conformational states. These conformational fluctuations are called channel gating. The primary experimental source of information about the channel gating phenomena is a patch clamp method [1]. It allows for recording a single-channel activity in the form of time series of ionic currents (Figure 1) at controlled conditions. Depending on an ion current amplitude, open and closed channel states are recognized. The main characteristics of the exemplary signal, presented in Figure 1, are common for most ion channel types.

The Markovian diagrams represent the standard kinetic model of these conformational changes with specific interconnections between the particular states and the respective transition probabilities [3,4,5]. In the simplest case, only one open and one closed state can represent channel activity, but frequently, more a sophisticated approach involving several open/closed states is addressed. The Markovian approach to model gating dynamics remained the most popular throughout the years [6]. It allows us to correctly describe many kinetic characteristics of the empirical system, such as the open-state probability or dwell-time distributions of open and closed channel states. Nevertheless, the dynamical properties of channel gating still require clarification in many aspects. The ion current sequences are the largest source of information about the channel’s system dynamics. The patch clamp recordings require appropriate data analysis methods, considering their highly complex characteristics and nonlinear properties [7,8].

Quantifying the physical system complexity is a tricky task. Ludwig Boltzmann historically introduced a measure of the number of possible states for the microscopic world. He connected it to Rudolf Clausius’ concept of entropy and the irreversibility of macroscopic processes. Entropy lies at the heart of the second law of thermodynamics, which states that entropy cannot decrease with time for isolated systems, which implies its maximum for the equilibrium state. Entropy is a thermodynamic property that can measure the amount of thermal energy in a system that is unavailable for doing useful work. It is now associated with disorder, randomness, or uncertainty, rather than the actual state-counting process. In such a case, it can serve as a measure of complexity—the more complex the system is, the higher entropy possesses. In 1948, Claude Shannon introduced the measure for missing information as an analog to the thermodynamic entropy [9]. Since then, information entropy has become a key concept for information theory. It has gained considerable popularity and effectiveness among the range of techniques that can be applied in the context of biological signals. A phenomenon of entropy measures is associated with its ability to characterize the rate of creation of valuable information in a dynamical system, identifying the level of uncertainty or the possibility of an indirect description of the number of available states, which can have a direct impact on many biological aspects [10]. The different kinds of entropy measures, in the forms of Shannon, Kolmogorov, approximate, or sample entropy, are involved in the analysis of electrophysiological signals, including cardiac rate variability [11,12], electromyography (EMG) [13], and electroencephalography (EEG) [14], to name but a few.

The ion channel signal reflects the complexity of the channel’s switching among its available states. Only a few reports have addressed the entropy-based patch clamp data analysis [2,15,16]. In [15], the values of the sample entropy of the signals, which characterized the large conductance calcium-activated potassium (BK) channel’s activity, were investigated in the context of effects of membrane strain and the possible changes of membrane morphology after a series of suction impulses. The work [16] describes the utilization of information entropy in distinguishing the patch clamp signals of mitochondrial BK channels (mitoBK) obtained for different cell types. In [2], the authors engaged the idea of entropy in the classification of mitoBK channels activity at changing experimental conditions (voltages). In addition, the authors used multiscale entropy to select the optimal sampling frequency rate of the ion current recordings.

Deterministic forces and stochastic thermal fluctuations shape the single-channel activity. In the mentioned works, entropy values were based only on experimental ion channel recordings. The complexity description gave combined characteristics of the signal, being both shaped by the deterministic interactions associated with protein–protein, protein–ligand, or protein–lipid interactions, as well as the thermal fluctuations of the helices forming the channel and its membrane surroundings. It is challenging to extract information about the relative impact of deterministic forces and stochastic thermal fluctuations on channel gating and signal entropy from the experimental data. Therefore, implementing the information entropy for the patch clamp recordings analysis has been based only on finding the differences in signal complexity with appropriately changed experimental conditions. In this work, we decided to go a step further and provide a more detailed description of how the properties of conformational space and the number of possible states of the channel influence entropy.

To that aim, we use data obtained in a simulation of a relatively simple Markovian channel gating model and investigate the effects of the changes in conformational diffusion space parameters on signal complexity. The investigated parameters will refer to the channel’s energetic landscape that governs the open–closed fluctuations in a confined space. Moreover, we decided to provide a signal description using different entropy measures. Our choice is concentrated on two leading groups of entropy features. We consider the standard Shannon information entropy and its frequency-based analog, in the form of spectral entropy. These two measures are based on probability distributions or power spectral density functions, and the interpretation of their values is limited to the statistical description of data. For the comprehensive characterization of ion channel activity, we decided to also select the specific kinds of entropy that can investigate the information rate considering the system’s dynamics. Sample entropy (SampEn) is one such measure, with proven effectiveness in biological signal classification [17] and the analysis of EEG and EMG signals [18]. However, popular entropy measures can sometimes underestimate the valuable information in complex data sets. The slope entropy, for instance, has a higher discriminating power in application to complex and numerically demanding data [18]. Since its introduction in 2019, the slope entropy algorithm has gained popularity for biological time series analysis. Moreover, the measure of slope entropy was successfully applied in fever diagnoses [19]. It was also a valuable feature in the machine learning ECG signal classification [20]. The method has been used for other types of signals, such as bearing fault signals [21] and ship-radiated noise signals [22].

Regarding the effectiveness of these techniques, we decided to set the slope and sample entropy results together to test the possible scenarios related to the potential change of information loss in such a complex ion channel system. In particular, we will test the response to the changes in the energetic landscape of conformational states (corresponding to the stability of available channels’ open and closed conformations) or the relative noise intensity (temperature).

## 2. Methods and Model

### 2.1. Information Analysis

The original Shannon idea of the signal description by its information content was later extended to new concepts of complexity measures, such as Rényi [23], spectral [24], Kolmogorov [25], approximate [26], sample [27], permutation [28], fuzzy [29], phase [30], and slope [18], to name but a few. It is now broadly used in almost all fields of science; see [31,32,33,34] and references therein. Here, we will focus on four selected quantifiers.

#### 2.1.1. Time and Frequency Domains

On average, the entropy of a signal *X* is related to the spectrum of the *k* potential states. For the probability distribution, p(X) of all possible states characterized by the probability $p_{k}$ of the *k*–th state, the **Shannon entropy** $H_{X}$ is given by the average of the logarithm of the distribution

(1) $$H_{X}=-\sum_{k}p_{k}\log\left(p_{k}\right)$$

with $\sum_{k}p_{k}=1$. For any experimental signal, the simplest way to determine a sample probability distribution is to take the data and determine its frequency, for example, by calculating the histogram and normalizing it by the total number of samples.

**Spectral entropy** (S) is the frequency-domain analog of the time-domain characteristics presented above. The power spectrum density (PSD) plays the role of the probability $p_{k}$. The spectral entropy is given in terms of the normalized PSD $Q\left(f\right)=PSD\left(f\right)/\sum_{f}PSD\left(f\right)$

(2) $$H_{f}=-\frac{1}{\log(F)}\sum_{f}Q\left(f\right)\log Q\left(f\right)$$

where *F* stands for the number of all frequency components. PSD is usually estimated with a standard (fast) Fourier transform. Because the frequency components are linearly independent, this measure is not sensitive to signal nonlinearities.

#### 2.1.2. Phase-Space Methods

The methods for estimating information loss based on probability distribution or PSD are easy to apply, although they lose some critical features of usual complex and nonlinear dynamics. For instance, $H_{X}$ would not consider the possible autocorrelation, and $H_{f}$ will evaluate only the linear features. The system’s dynamic diversity should be a key component for the typical analysis. Kolmogorov developed the method to introduce the effect of the system’s dynamical changes in 1950’. The metric entropy uses correlation integral for a dynamical complexity estimation [35,36]. The actual method used in calculations looks for the number of repetitions of the vector patterns in the entire signal. As the original concept addresses the infinite vectors and data lengths, but real-life experiments produce only finite data lengths, the technique to determine the changing system complexity was first proposed by Pincus as approximate entropy [26]. It quantifies the tendency of signal chunks to repeat.

The weakness of the original formulation of the algorithm for obtaining metric entropy (inclusion of self-matches) was corrected later, in terms of **sample entropy** (SampEn) [27]. For the time series $X={\left\{x_{i}\right\}}_{i=1}^{N}$, which consists of *N* data points, a set of vectors $U_{m}\left(i\right)=\{{x_{i},\ldots ,x_{i+m-1}\}}_{i=1}^{N-m+1}$ represents the *m* consecutive values of a series, starting with the i−th data point. The difference between two sets $U_{m}\left(i\right)$ and $U_{m}\left(j\right)$ is taken as the maximum metric

(3) $$d\left[U_{m}\left(i\right),U_{m}\left(j\right)\right]=\max_{k=0,\ldots ,m-1}(|x\left(i+k\right)-x\left(j+k\right)|).$$

The probability of an exact match of *m* consecutive vector elements is basically zero for the experimental data. To confirm the similarity between two sample vectors $U_{m}\left(i\right)$ and $U_{m}\left(j\right)$, one has to introduce the tolerance threshold *r* heuristically. Usually, it is taken between 10% and 20% of the standard deviation σ of the signal values [37]. If the absolute difference of any pair of the components is larger than the chosen similarity criterion (distance threshold) $d[U_{m}\left(i\right),U_{m}\left(j\right)]>r$, the vectors are not similar.

The probability $C_{i}^{m}\left(r\right)$ that the *i*–th template vector is close to any other is given by

(4) $$C_{i}^{m}\left(r\right)=\frac{n_{i}^{m}\left(r\right)}{N-m}$$

where $n_{i}^{m}\left(r\right)$ is a number of other *j*–vectors (j=1,…,i−1,i+1,…,N−m) that are close enough to the pattern *i*–vector. The average over the all pattern vectors $U_{m}\left(i\right)$ constitutes the probability $C^{m}\left(r\right)$ that any two vectors are within *r* of each other

(5) $$C^{m}\left(r\right)=\frac{1}{N-m+1}\sum_{i=1}^{N-m+1}C_{i}^{m}\left(r\right).$$

The negative logarithm of the conditional probability that two patterns that are similar for *m* points remain akin for m+1 points defines the sample entropy

(6) $$\mathrm{SampEn}\left(m,r,N\right)=-\log\left[\frac{C^{m+1}\left(r\right)}{C^{m}\left(r\right)}\right]$$

For the above calculations, j≠i. In the following, the values of embedding dimension m=4 and r=0.2σ have been used.

The SampEn algorithm considers the intrinsic states of the system dynamics and uses the template vectors of size *m* as the patterns. It does not, however, reflect the point-to-point changes through the signal. Such issues are embedded in the idea of the **slope entropy** algorithm, which is based on the signal amplitude and can be characterized by the following steps. Similarly to the SampEn algorithm, the time series $X={\left\{x_{i}\right\}}_{i=1}^{N}$ is divided into *k* sub-sequences $U_{m}\left(i\right)$. The two additional threshold parameters δ and γ are arbitrarily defined to assign the different symbol patterns that characterize the vertical increments between consecutive samples. Typically, γ=1 and δ=0.001 correspond to the $45^{\circ }$ and $0.05^{\circ }$ slope of the line connecting two successive points, respectively. In the standard version of the algorithm, the division covers the five basic patterns: {+2,+1,0,−1,−2}. If $x_{i}\geq x_{i-1}$ + γ, and the assigned symbol is +2. For the $x_{i-1}+\delta \leq x_{i}<x_{i-1}+\gamma$, the symbol +1 is used. For tiny increments $x_{i-1}-\delta <x_{i}<x_{i-1}+\delta$, the assumed symbol is 0. Considering the mirror image of symbols +1 and +2 for analogical negative decrements, we can assign the obtained amplitude differences to the patterns −2 and −1 for γ=−1 and δ=−0.001, respectively.

There are $5^{m-1}$ types of pattern sequences with the number of each type ${\left\{k_{i}\right\}}_{i=1}^{n}$. For *k* unique template vectors, the probability of occurrences of *i*–th pattern is given by $P_{i}=\frac{k_{i}}{k}$. Using the standard entropy form, the slope information measure reads

(7) $$\mathrm{SlopEn}=-\sum_{i=1}^{n}P_{i}\log P_{i}$$

The method to keep the SlopEn values within the usual [0,1] interval was proposed recently [38]. In this work, as suggested, we would use the minimum heuristic bound and the analytic maximum to normalize the calculated values.

Shannon analyzed the electronic signal transmission, so his definition of entropy naturally uses two as the base of logarithm and, in turn, *bits* as the unit of the information loss. In this work, we avoid including extra constants and use a natural logarithm, which results in natural units or *nats*.

### 2.2. Ion Channel Current Dynamics

The functional dynamics of the ion channel are relatively simple. The channel is either conducting or not. In other words, the current is either flowing through the channel or not. In such a case, the channel can remain in either an open or closed state. The simplest possible scenario assumes one open state ($O_{1}$) and one close state ($C_{2}$), so the channel can only alternate between the two states [39] with the transition rates $k_{1-2}$ and $k_{2-1}$

$$O_{1}\underset{k_{2-1}}{\overset{k_{1-2}}{⇌}}C_{2}$$

The time the channel spends in one of the states is governed by the exponential distribution characterized by the average dwell time in the specific state τ=1/k [39].

(8) $$f\left(t\right)=\frac{1}{\tau }\exp\left(-\frac{t}{\tau }\right)$$

For single-channel kinetics, we can estimate the average voltage rather precisely, although the intrinsic value will always fluctuate. The statistical characteristics of such fluctuations are well-known [40].

The actual dynamics can and is usually much more complex. We can often distinguish several open ($O_{1},\ldots ,O_{i}$) and closed ($C_{i+1},\ldots ,C_{n}$) states [41]. The transitions between states are not always allowed. More complex distributions can describe the fluctuations of the recordings of ion current in a given state. As an example, one can mention α−stable Levy distribution. On top of that, the fluctuations can exhibit considerable correlations. Despite the rich complexity of the experimental data and the actual biosystems, there is no necessity to address full, and often highly complicated, mechanisms to describe some fundamental problems. Here, we model such issues as simply as possible. We want to ask how the modification in the energetic landscape of conformational states influences the total complexity of the system and how the chosen measures will react to such changes. We also would test the model and the measures mentioned above against the temperature. We will probe the proposed characteristics with a system with artificially changed complexity.

### 2.3. Stochastic Model

We model channel gate dynamics with stochastic evolution equations [42,43,44]. We map the ion channel dynamics onto the overdamped Brownian particle moving in confined potential and driven by thermal noise. The dynamics of such a particle is governed by the Langevin equation

(9) $$\Gamma \dot{x}\left(t\right)=-V^{\prime }\left(x\right)+\sqrt{2\Gamma D}\xi \left(t\right)$$

where the prime denotes a differentiation with respect to the argument of V(x) and the dot means a differentiation with respect to time *t*. Thermal fluctuations are modeled by the zero-mean δ-correlated Gaussian white noise ξ. This noise term obeys the Einstein relation with the noise correlation given by 〈ξ(t)ξ(s)〉=δ(t−s). Two constants, Γ and D=kT, stand for the friction coefficient and noise intensity, respectively. Capital *T* is the temperature, and *k* symbolizes the Boltzmann constant. Without the loss of generality, we will keep the friction coefficient equal to one (Γ=1) in what follows.

The particle can take two distinct states corresponding to the ion current flowing (open state, $x_{O}$) and not flowing through the channel (closed state, $x_{C}$). For the confined potential, we consider a simple quadratic form

(10) $$V\left(x\right)=b{\left(x-x_{s}\right)}^{2}$$

with minimum located at the $x_{s}=x_{O}$ or $x_{C}$. The operational state randomly changes from open to closed, according to dwell times distribution (8). Here, it corresponds to a change of the potential minimum $x_{s}$. The parameter *b* defines the slope of the potential walls and, in turn, influences the localization of the particle and, as such, mimics the change of the energetic landscape of conformational states. For b<1, the potential walls are less steep, which allows the particle to move more freely around the minimum. If, however, b>1, the steepness of the walls grows, and travel further from the minimum requires higher energies.

**Figure 2:**
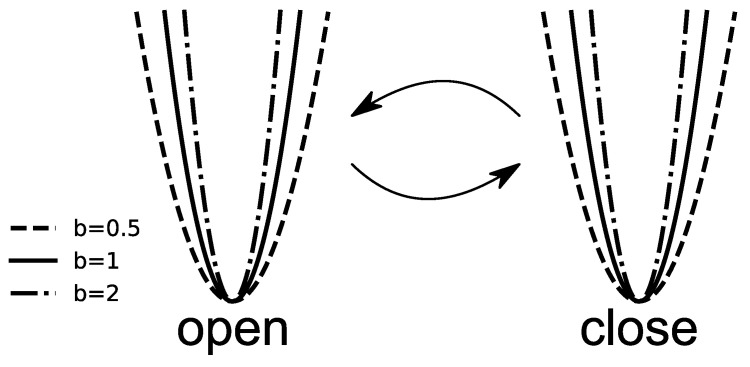
The schematic representation of the ion channel activity modeled utilizing the Langevin Equation (9). The random switches between the states represent the simplified Markov kinetics. The steepness of the potential walls, ruled by the parameter *b*, mimics the change of the energetic landscape of ion channels. In the simplest case, only one open and one closed state is assumed. Intra-well dynamics is driven by the δ-correlated Gaussian white noise of zero average.

The model landscape is schematically depicted in Figure 2. We performed numerical simulations of the Langevin Equation (9) using an Euler algorithm with a step size of $10^{-2}$. We always draw the first state uniformly. The particle would sit in one of the potential wells (states) for a certain period $\tau_{1}$ drawn from the dwell-time distribution of the current state. Intra-well motion is driven solely by the temperature-dependent Gaussian white noise. After time $\tau_{1}$, it will change the state to a new permitted one, again for a random time $\tau_{2}$, and again execute the random motion inside the potential well. This sequence of randomly drawing the next (permitted) state and performing inside the selected potential well will be repeated until the simulation’s total time ($T_{t}$, here =100) passes. In such a case, we stop the generation of the first trajectory and repeat the process a minimum of 100 times. It gives us statistics of 100 independent runs, each consisting of 100.000 data points. Further enlargement of the number of runs or data points keeps the statistics the same and only unnecessarily increases the computational cost.

## 3. Results

**Figure 3:**
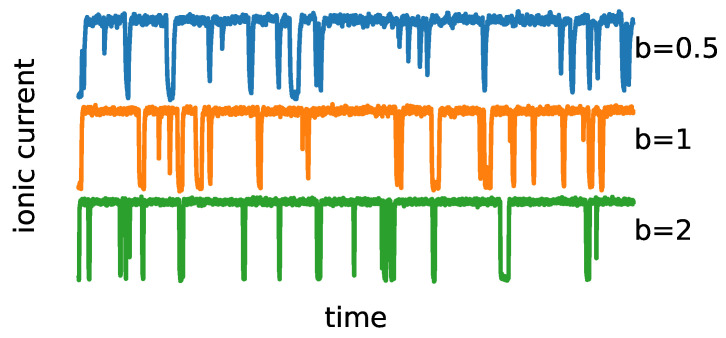
Sample trajectories (ionic currents) generated using Langevin Equation (9) and the simple scheme of one open $O_{1}$ and one closed $C_{2}$ states. The average dwell times for open and closed states are ${\hat{\tau }}_{1}=1$ and ${\hat{\tau }}_{2}=10$, respectively. In such a case, the only allowed transitions happen between the $O_{1}$ (**bottom**) and $C_{2}$ (**top**) states. Other parameters read D=0.01, b=0.5,1,2.

The representative trajectories obtained by model simulation are presented in Figure 3. As one can see, the overall signal characteristics mimic the patch clamp recordings. The effects of the steepness of potential wells (*b*) can be observed in the form of a varying range of the single-channel current amplitudes, which correspond to the open and closed states, at a fixed noise level (determined by the *D* parameter). Thus, the model is anticipated to enable the imitation of the experimental ionic current distributions after appropriate parameter optimizations.

The following results of the numerical simulations are presented for the most straightforward possible setup with only two allowed states—one open $O_{1}$ and one closed $C_{2}$—as the proposed complexity measures are not sensitive to the number of channel states (if they all exhibit the same characteristic O/C conductance). Regardless of the ion channel kinetics’ number of states, the ionic current would always have the same value in any open (or similarly closed) states. The possibility of channel current sublevels is not considered here, since it is not very often exhibited in biological systems. The proposed measures are all based on the values in the data train, and since there is no difference in values between, say, the states $O_{1}$ and $O_{2}$, one will not see the change in entropy.

### 3.1. Temperature Influence

Calculating the information entropy for white Gaussian noise, for which all the possible states are allowed, would result in the maximum entropy value [31]. In terms of Langevin dynamics, this situation describes the free Brownian particle with no potential force present. In such a situation, with no other energy scale to compare to, the effective information loss will be the same, regardless of the noise intensity (heat bath temperature), and the function of (any) entropy *versus D* will remain constant for any value of *D*.

If, however, one would reduce the ability of travel for the particle by placing it inside the entropic potential, the whole picture would change. The Brownian particle cannot pass through the barriers of the proposed quadratic potential and remains located around the potential minimum, regardless of the open or closed state. The increasing temperature will increase the thermal energy available for the particle. This, in turn, would enable the particle to travel further against the gradient and visit distant locations in the potential well, causing global complexity to rise.

**Figure 4:**
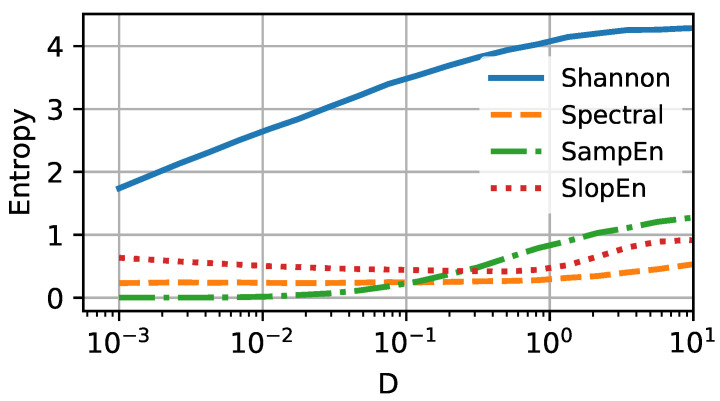
Four selected entropy measures—Shannon (solid blue line), Spectral (dashed orange line), Sample (dashed-dotted green line), and Slope (dotted red line)—presented *versus* the noise intensity *D* (or temperature) for the simplest setup. Steepness parameter b=10.

We can confirm the expectations for three of the four selected entropy measures. In Figure 4, the four selected entropy measures (Shannon, spectral, sample, and slope) are shown *versus* the noise intensity *D*. In this scenario, the particle deals with the energy barrier, which causes the particle to sit around the potential minima separately for the open and closed states. The value of Shannon entropy is constantly rising with increasing thermal energy *D*, to the point where it saturates for the intensity D≃2. Spectral and sample entropies remain constant for a broad selection of intensities *D*. The former is slightly more sensitive to temperature change and increases around D≃0.05. The latter rises visibly for D≃1. The slope entropy behaves somewhat differently and reveals a non-monotonic tendency with increasing *D*. Initially, (D∈[0.001,1]) drops slightly. Near unity, it reaches a minimum and then starts to increase. The existence of extrema in the complexity measure is usually responsible for the occurrence of critical phenomena. Here, we could not find any such behavior in the vicinity of D=1, and the presence of the minimum remains the puzzle. As the SlopEn algorithm rather aggressively affects the information carried in the data, this effect may be caused by the algorithm’s structure.

**Figure 5:**
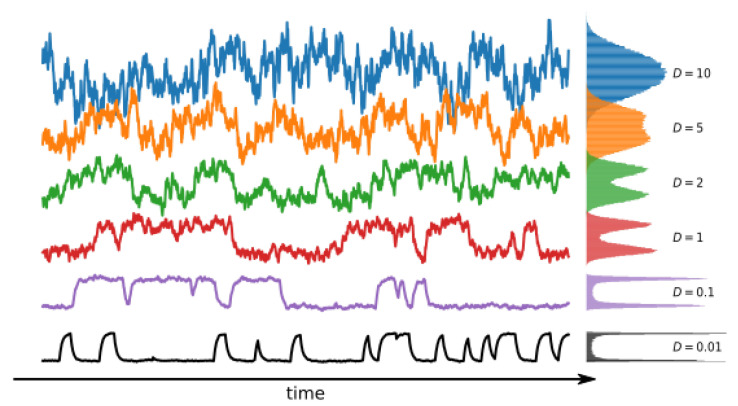
Sample trajectories of Brownian particle motion, which mimics the ionic current generated with the Equation (9) for six values of the noise intensity (bottom to top) D=0.01, 0.1, 1, 2, 5, 10. On the rhs, the corresponding histograms are plotted. Please note the vanishing two-state characteristics for the two highest values of D=5,10. The landscape parameter is set to b=10. Each trajectory consists of 2000 data points.

Above the noise intensity D≃5, the structure of the signal is no longer similar to the one registered in the patch clamp experiment, and the analysis becomes meaningless. In Figure 5, we present the trajectories (ionic currents) for the selected values of the noise intensity, together with the corresponding distributions (histograms). Please note the vanishing separation of states in the distributions for higher noise intensities (D=5 and 10).

### 3.2. Localization

**Figure 6:**
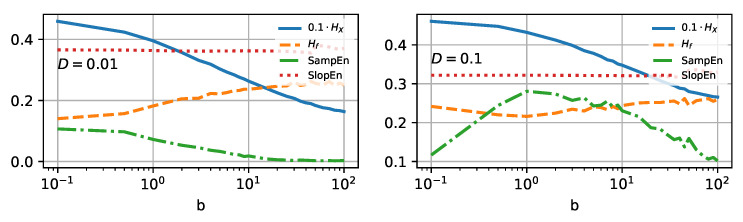
Four selected entropy measures—Shannon (solid blue line), Spectral (dashed orange line), Sample (dashed-dotted green line), and Slope (dotted red line) presented *versus* the steepness factor *b* (or landscape parameter) for the simplest setup. The **left** and **right** panels correspond to noise intensities D=0.01 and 0.1, respectively. Shannon entropy has been scaled ten times down for visual clarity.

Reducing the ability to travel should mean reducing the information complexity and randomness of the system, as we limit the number of possible states of the Brownian particle. In our case, the most direct possibility to influence the localization will be adjusting the landscape parameter *b* of the potential (10). In Figure 6, we present all four selected entropy measures (colors and line types stay the same as in Figure 4).

**Figure 7:**
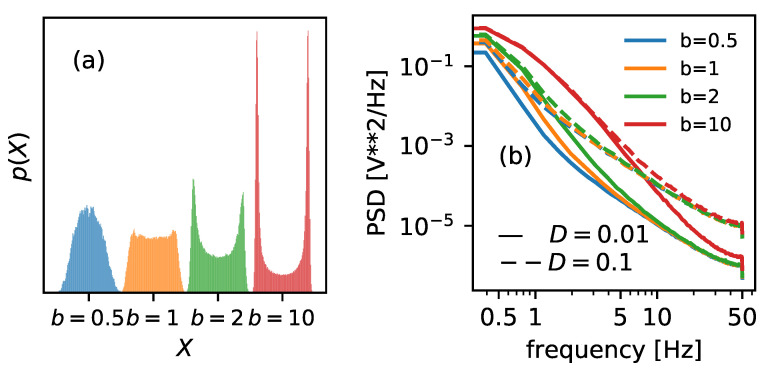
(**a**) The distributions of the positions of the Brownian particle, together with the corresponding potential shapes. (**b**) The power spectrum densities of the Brownian particle mimicking ionic current presented for two different noise intensities D=0.01 (solid lines) and D=0.1 (dashed lines). Both characteristics are plotted for four values of the landscape parameters b=0.5 (blue), 1 (orange), 2 (green), and 10 (red).

Increasing the parameter *b* causes the narrowing of the potential walls; see Figure 2 for details. It will also cause the probability distribution of possible states to narrow. In Figure 7a, the distributions of the positions of the Brownian particle are plotted. By inspecting the graphs, one can expect the descending Shannon entropy measure, which depends on the number of possible states (the width of the histograms).

The narrower the distribution, the fewer states accessible, and the lower the Shannon entropy, as it is solely given as a function of the distribution. Therefore, it is not surprising that the Shannon entropy curve decreases monotonically as a function of the increasing value of *b*, regardless of the scaled temperature considered—cf. solid blue lines on both panels of Figure 6.

Spectral entropy, on the other hand, behaves somewhat unexpectedly. The PSD inspection can explain its increase—cf. Figure 7b. The area under the curve increases with parameter *b*, regardless of the noise intensity. This, in turn, should increase entropy measure based on these characteristics—cf. the orange dashed line in Figure 6. However, for higher noise intensity D=0.1, the tendency is not monotonic, and the $H_{f}$ assumes a minimum in the vicinity of b=1.

Sample entropy (dashed-dotted green line in Figure 6) is a decreasing function of the growing landscape parameter for low noise intensity D=0.01, the behavior known for the classical $H_{X}$. The lower complexity is somewhat expected for an algorithm based on estimating the frequency of the possible template vectors. The narrower the potential wells, the lower number of unique patterns. For higher scaled temperature, D=0.1, SampEn surprisingly reveals a maximum in the vicinity of b=1. Similarly to $H_{f}$, one can expect a somewhat critical phenomenon around that value [45,46].

In contradiction to the temperature dependence, slope entropy remains constant for any of the examined values of *b*. It suggests that increments of ionic currents over time are statistically identical for potential conformational change. Still, one can notice this measure’s lower, although still constant, value for D=0.1. Based on the increments, rather than the values themselves, the information loss estimate remains insensitive to localization changes.

## 4. Discussion

The movement of ions across a biological membrane through channels is passive, meaning it occurs without the input of energy from the cell. The electrostatic potential gradient and the difference in ion concentration between both sides of the membrane drive this movement. Active ion transport is the movement of ions across a biological membrane against the gradient from an area of lower concentration to an area of higher concentration. This process requires energy input from the cell, often in the form of adenosine triphosphate. The energy input pumps ions across the membrane against their gradient, creating a concentration and an electrochemical gradient. Active ion transport plays a crucial role in maintaining ionic balance and cell homeostasis. Examples of active ion transport include the sodium–potassium pump and the proton pump. Most works would model one of the mechanisms employing continuum or polarizable models [44] and references therein.

In this work, we focused solely on modeling the ionic current without first assuming the type of transport across cell membranes. We built a simple model with two-dimensional parameter space {D=kT,b} by means of the overdamped Langevin equation. The main goal was to describe the potential effects of temperature and the channel’s energetic landscape for conformational changes on the ionic current’s complexity without going into unnecessary model details. To accurately describe the information entropy of a biosystem, we used two classic measures of randomness, Shannon and spectral entropies, as well as two relatively new quantifiers that are very successful in the classification of biological signals, sample and slope entropies.

The Shannon entropy ($H_{X}$) exhibits typical, predictable behavior when we force the system to change the number of possible states, either by increasing the temperature ($H_{X}$ is also increasing) or by forcibly increasing the location of the Brownian particle ($H_{X}$ is decreasing). The spectral entropy behaves normally with temperature changes and increases as we heat the system. It is slightly less predictable for changes in the location of the Brownian particle. For particles with a reduced possibility of random motion, it is expressed in increased entropy based on the PSD for relatively low temperatures. For higher temperatures, the randomness characteristic shows a minimum for a specific value of the shape of the potential well.

Complexity measures based on system state vectors calculated for raw values directly from the data seem less predictable than classical counterparts. The sample entropy for the temperature dependence behaves similarly to the classical Shannon measure and increases with the temperature, showing an increase in the unpredictability of the biosystem. Additionally, similar to $H_{X}$, it reacts to the restriction of the particle’s motion, showing a decrease in entropy for particles restrained by the possibility of visiting states distant from the minima, at least for lower temperatures. For higher temperatures, where the position close to the minimum is not so clear, and the particles can travel much higher along the potential walls, SampEn shows a maximum for the selected potential configuration. There may be a critical phenomenon for the dynamics, although we have yet to find any during careful examination of the trajectories and state vectors. Slope entropy exhibits slightly different behavior with increasing temperature. We find a shallow minimum in the *D* function for this characteristic. It seems surprising that this measure does not respond to the increase in the location of the Brownian particle. It can be explained with the same probability of occurrence of increments of the ion current value.

## 5. Conclusions

The effects of two parameters affecting single-channel gating on the complexity of the corresponding time series of ionic currents have been analyzed. In particular, we analyzed the effects of temperature and steepness of potential wells separating a channel’s open and closed functional states on the values of signals’ information entropy. The first analyzed parameter, scaled temperature *D*, is mostly related to the signal-to-noise ratio in the experimental patch clamp recordings. The second one, *b*, describes the effects of energetic and spatial constraints on the channel gate dynamics. Due to the fact that both aspects are hardly controllable in the biological system, our studies are performed on the simulated data, where the mechanism is ruled by the popular two-state Markovian approach [1]. The simulated current trajectories allow us to directly observe the effects of thermal fluctuations, which represent the perturbations within the recordings of the single-channel activity, as well as the gating constraints on the characteristics of the signal representing the single-channel activity. The signals’ entropy was calculated by four different measures (Shannon, spectral, sample, and slope entropies), and the obtained results allow us to observe the changes in the signals’ complexity.

The increase in temperature should be gathered by the increase of entropy. In turn, the higher restrictions for the conformational diffusion, represented by the *b* parameter, are anticipated to lower the complexity. These predictions are based on the thermodynamic considerations of the possible availability of states and the complexity of the channel current values at planned conditions. Our results allow us to conclude that only the Shannon definition provides an entropy measure that enables us to directly reflect the relations discussed above. Thus, the reliability and performance of the Shannon entropy in the analysis of time series describing the single-channel gating dynamics can be highly rated and recommended. The results obtained by the other entropy measures are more tricky or precarious. Therefore, their utility in ion channel-dedicated studies needs further examination.

Additional current states found in the literature (as ”sublevels”) can be incorporated to make the model more universal. Including additional open and closed states of different characteristic conductance could increase the randomness of the model, which can better imitate the recorded currents flowing through some ion channel types in the biological membranes. This can lead to a better understanding of ion transport processes and how they contribute to the functioning of biological membranes.

## Data Availability

The data have been simulated with the original software. Please get in touch with the corresponding author.

## Abbreviations

The following abbreviations are used in this manuscript:

BK	Large conductance calcium-activated potassium channels
ECG	Electromyography
EEG	Electroencephalography
$H_{f}$	Spectral entropy
$H_{X}$	Shannon entropy
PSD	Power spectral density
SampEn	Sample entropy
SDE	Stochastic differential equation
SlopEn	Slope entropy

## References

[1] Sakmann B. (2013). Single-Channel Recording.

[2] Machura L., Wawrzkiewicz-Jałowiecka A., Bednarczyk P., Trybek P. (2022). Linking the sampling frequency with multiscale entropy to classify mitoBK patch-clamp data. Biomed. Signal Process. Control.

[3] Colquhoun D., Hawkes A.G. (1977). Relaxation and fluctuations of membrane currents that flow through drug-operated channels. Proc. R. Soc. Lond. Ser. B Biol. Sci..

[4] Colquhoun D., Hawkes A. (1981). On the stochastic properties of single ion channels. Proc. R. Soc. Lond. Ser. B Biol. Sci..

[5] Colquhoun D., Hawkes A.G. (1982). On the stochastic properties of bursts of single ion channel openings and of clusters of bursts. Philos. Trans. R. Soc. Lond. Ser. B Biol. Sci..

[6] Linaro D., Giugliano M. (2022). Markov models of ion channels. Encyclopedia of Computational Neuroscience.

[7] DeFelice L.J., Isaac A. (1993). Chaotic states in a random world: Relationship between the nonlinear differential equations of excitability and the stochastic properties of ion channels. J. Stat. Phys..

[8] Liebovitch L.S., Todorov A.T. (1996). Using fractals and nonlinear dynamics to determine the physical properties of ion channel proteins. Crit. Rev. Neurobiol..

[9] Shannon C.E. (1948). A mathematical theory of communication. Bell Syst. Tech. J..

[10] Gao J., Hu J., Tung W.W. (2012). Entropy measures for biological signal analyses. Nonlinear Dyn..

[11] Sharma K., Sunkaria R.K. (2023). Novel multiscale E-metric cross-sample entropy-based cardiac arrhythmia detection and its performance investigation in reference to multiscale cross-sample entropy-based analysis. Signal Image Video Process..

[12] Yan C., Li P., Yang M., Li Y., Li J., Zhang H., Liu C. (2022). Entropy analysis of heart rate variability in different sleep stages. Entropy.

[13] Trybek P., Nowakowski M., Salowka J., Spiechowicz J., Machura L. (2018). Sample entropy of sEMG signals at different stages of rectal cancer treatment. Entropy.

[14] Wang Y., Liang Z., Voss L.J., Sleigh J.W., Li X. (2014). Multi-scale sample entropy of electroencephalography during sevoflurane anesthesia. J. Clin. Monit. Comput..

[15] Wawrzkiewicz-Jałowiecka A., Trybek P., Machura Ł., Dworakowska B., Grzywna Z.J. (2018). Mechanosensitivity of the BK channels in human glioblastoma cells: Kinetics and dynamical complexity. J. Membr. Biol..

[16] Wawrzkiewicz-Jałowiecka A., Trybek P., Machura Ł., Bednarczyk P. (2021). Dynamical diversity of mitochondrial BK channels located in different cell types. Biosystems.

[17] Aboy M., Cuesta-Frau D., Austin D., Mico-Tormos P. Characterization of sample entropy in the context of biomedical signal analysis. Proceedings of the 2007 29th Annual International Conference of the IEEE Engineering in Medicine and Biology Society.

[18] Cuesta-Frau D. (2019). Slope entropy: A new time series complexity estimator based on both symbolic patterns and amplitude information. Entropy.

[19] Cuesta-Frau D., Dakappa P.H., Mahabala C., Gupta A.R. (2020). Fever time series analysis using slope entropy. Application to early unobtrusive differential diagnosis. Entropy.

[20] Fuadah Y.N., Lim K.M. (2022). Optimal Classification of Atrial Fibrillation and Congestive Heart Failure Using Machine Learning. Front. Physiol..

[21] Shi E. (2022). Single Feature Extraction Method of Bearing Fault Signals Based on Slope Entropy. Shock Vib..

[22] Li Y., Gao P., Tang B., Yi Y., Zhang J. (2022). Double feature extraction method of ship-radiated noise signal based on slope entropy and permutation entropy. Entropy.

[23] Rényi A. (1961). On measures of entropy and information. Proceedings of the Fourth Berkeley Symposium on Mathematical Statistics and Probability, Volume 1: Contributions to the Theory of Statistics.

[24] Powell G., Percival I. (1979). A spectral entropy method for distinguishing regular and irregular motion of Hamiltonian systems. J. Phys. Math. Gen..

[25] Grassberger P., Procaccia I. (1983). Estimation of the Kolmogorov entropy from a chaotic signal. Phys. Rev. A.

[26] Pincus S.M. (1991). Approximate entropy as a measure of system complexity. Proc. Natl. Acad. Sci. USA.

[27] Richman J.S., Moorman J.R. (2000). Physiological time-series analysis using approximate entropy and sample entropy. Am. J. Physiol.-Heart Circ. Physiol..

[28] Bandt C., Pompe B. (2002). Permutation entropy: A natural complexity measure for time series. Phys. Rev. Lett..

[29] Chen W., Wang Z., Xie H., Yu W. (2007). Characterization of surface EMG signal based on fuzzy entropy. IEEE Trans. Neural Syst. Rehabil. Eng..

[30] Rohila A., Sharma A. (2019). Phase entropy: A new complexity measure for heart rate variability. Physiol. Meas..

[31] Semmlow J.L. (2008). Biosignal and Medical Image Processing.

[32] Natal J., Ávila I., Tsukahara V.B., Pinheiro M., Maciel C.D. (2021). Entropy: From thermodynamics to information processing. Entropy.

[33] Ribeiro M., Henriques T., Castro L., Souto A., Antunes L., Costa-Santos C., Teixeira A. (2021). The entropy universe. Entropy.

[34] Tsallis C. (2022). Entropy. Encyclopedia.

[35] Kolmogorov A.N. (1958). New Metric Invariant of Transitive Dynamical Systems and Endomorphisms of Lebesgue Spaces.

[36] Kolmogorov A.N. (1959). Entropy per unit time as a metric invariant of automorphisms.

[37] Costa M., Peng C.K., Goldberger A.L., Hausdorff J.M. (2003). Multiscale entropy analysis of human gait dynamics. Physica A.

[38] Cuesta-Frau D., Kouka M., Silvestre-Blanes J., Sempere-Payá V. (2023). Slope Entropy Normalisation by Means of Analytical and Heuristic Reference Values. Entropy.

[39] Geng Y., Magleby K.L. (2015). Single-channel kinetics of BK (Slo1) channels. Front. Physiol..

[40] Zheng J., Trudeau M.C. (2015). Handbook of Ion Channels.

[41] Magleby K., Song L. (1992). Dependency plots suggest the kinetic structure of ion channels. Proc. R. Soc. Lond. Ser. B Biol. Sci..

[42] Ball F.G., Rice J.A. (1992). Stochastic models for ion channels: Introduction and bibliography. Math. Biosci..

[43] Sigg D. (2014). Modeling ion channels: Past, present, and future. J. Gen. Physiol..

[44] Maffeo C., Bhattacharya S., Yoo J., Wells D., Aksimentiev A. (2012). Modeling and simulation of ion channels. Chem. Rev..

[45] Frauenfelder H. (1987). Function and Dynamics of Myoglobin a. Ann. N. Y. Acad. Sci..

[46] Chialvo D.R. (2018). Life at the edge: Complexity and criticality in biological function. Acta Phys. Pol. B.

